# Bovine Embryo-Secreted microRNA-30c Is a Potential Non-invasive Biomarker for Hampered Preimplantation Developmental Competence

**DOI:** 10.3389/fgene.2019.00315

**Published:** 2019-04-05

**Authors:** Xiaoyuan Lin, Evy Beckers, Séan Mc Cafferty, Yannick Gansemans, Katarzyna Joanna Szymańska, Krishna Chaitanya Pavani, João Portela Catani, Filip Van Nieuwerburgh, Dieter Deforce, Petra De Sutter, Ann Van Soom, Luc Peelman

**Affiliations:** ^1^Department of Nutrition, Genetics and Ethology, Faculty of Veterinary Medicine, Ghent University, Merelbeke, Belgium; ^2^Department of Pharmaceutics, Faculty of Pharmaceutical Sciences, Ghent University, Ghent, Belgium; ^3^Physiology Group, Department of Basic Medical Sciences, Ghent University, Ghent, Belgium; ^4^Reproduction, Obstetrics and Herd Health, Ghent University, Merelbeke, Belgium; ^5^Department of Uro-Gynaecology, Faculty of Medicine and Health Sciences, Ghent University, Ghent, Belgium

**Keywords:** bovine embryos, secreted miRNAs, miR-30c, *CDK12*, cell cycle, DNA damage response, individual *in vitro* production

## Abstract

Recently, secreted microRNAs (miRNAs) have received a lot of attention since they may act as autocrine factors. However, how secreted miRNAs influence embryonic development is still poorly understood. We identified 294 miRNAs, 114 known, and 180 novel, in the conditioned medium of individually cultured bovine embryos. Of these miRNAs, miR-30c and miR-10b were much more abundant in conditioned medium of slow cleaving embryos compared to intermediate cleaving ones. MiR-10b, miR-novel-44, and miR-novel-45 were higher expressed in the conditioned medium of degenerate embryos compared to blastocysts, while the reverse was observed for miR-novel-113 and miR-novel-139. Supplementation of miR-30c mimics into the culture medium confirmed the uptake of miR-30c mimics by embryos and resulted in increased cell apoptosis, as also shown after delivery of miR-30c mimics in Madin-Darby bovine kidney cells (MDBKs). We also demonstrated that miR-30c directly targets Cyclin-dependent kinase 12 (*CDK12*) through its 3′ untranslated region (3′-UTR) and inhibits its expression. Overexpression and downregulation of *CDK12* revealed the opposite results of the delivery of miRNA-30c mimics and inhibitor. The significant down-regulation of several tested DNA damage response (DDR) genes, after increasing miR-30c or reducing *CDK12* expression, suggests a possible role for miR-30c in regulating embryo development through DDR pathways.

## Introduction

Many studies have indicated that the timing of cell division during the early embryonic stages is crucial for normal development and can be used as an indicator of embryo development competence (Fenwick et al., 2002; Zernicka-Goetz, 2002, 2006; Plusa et al., 2005; Hiiragi et al., 2006; Terriou et al., 2007). For example, the delay of cell division might be a consequence of chromosomal aberrations and DNA damage (Milewski et al., 2018) and slow cleaving embryos have a higher caspase activity in comparison to fast cleavers (Vandaele et al., 2007). In general, faster cleaving embryos have a significantly higher probability of reaching advanced developmental stages compared to slower cleaving embryos (Van Soom, 1997; Meirelles et al., 2004; Vandaele et al., 2006), while some studies also demonstrated that cleaving divisions that are too fast or too slow are indicative of poor embryo quality (Market Velker et al., 2012; Gutierrez-Adan et al., 2015). During these early embryonic stages, miRNA levels undergo dynamic changes (Mineno et al., 2006; Tang et al., 2007; Yang et al., 2008; Viswanathan et al., 2009; Goossens et al., 2013), indicating their potential role in embryonic development.

With this study we wanted to investigate if one or more miRNAs have potential as a non-invasive biomarker for preimplantation developmental competence according to cleavage patterns and blastocyst formation. It has been reported that miRNAs are not only localized intracellularly but also secreted via exosomes (Valadi et al., 2007). In addition, miRNAs have been reported to be transferable to other cells, and can be functional in the new location (Valadi et al., 2007; Sohel et al., 2013; Vilella et al., 2015). More specifically, they can be taken up into cells from the extracellular environment, leading to a corresponding endogenous miRNA increase in transfected cells. Recently, secreted miRNA expression was reported to correlate with developmental competence and sexual dimorphism in bovine (Kropp and Khatib, 2015; Gross et al., 2017) and human embryos (Rosenbluth et al., 2014). Although the precise mechanisms of miRNA release in the cellular environment are poorly understood, their selective secretion and high stability (resistant to RNase digestion and other harsh conditions) make miRNAs good candidates for use as biomarkers (Luo et al., 2009; Donker et al., 2012). Potential limitations for their use as biomarkers are their general low abundance, and the high sequence identity among family members.

miR-30 family members are involved in the regulation of p53-induced mitochondrial fission and cell apoptosis (Li et al., 2010). As a member of the miR-30 family, miR-30c has been shown to regulate the cell cycle and proliferation in human and mouse (Li et al., 2012; Quintavalle et al., 2013; Shukla et al., 2015; Liu et al., 2016). One of its potential targets as determined by our study is *CDK12* mRNA. *CDK12* is a protein kinase responsible for mature mRNA synthesis transcriptional elongation (Bartkowiak et al., 2010; Liang et al., 2015). This kinase has been reported to be crucial for the development of the inner cell mass in mouse embryos (Juan et al., 2016) and to maintain genomic stability as Cyclin K/*CDK12* complex (Blazek et al., 2011) through regulating DDR genes. In this study, we demonstrated that miR-30c is secreted and taken up by bovine embryos and functions as a negative regulator of cell growth by targeting *CDK12*, indicating that miR-30c can be considered as a promising biomarker for bovine early embryonic development. These findings may provide new insights into understanding the regulatory role of secreted miRNAs in the process of intercellular communication.

## Materials and Methods

### *In vitro* Embryo Production and CM Collection

All animal handlings were approved by the Ethical Committee of the Faculty of Veterinary Medicine (EC2013/118) of Ghent University. All methods were performed in accordance with the relevant guidelines and regulations. Bovine blastocysts were produced according to the previously used routine *in vitro* fertilization (IVF) methods in our lab (Wydooghe et al., 2014a). Briefly, ovaries were collected from the local slaughterhouse and processed within 2 h. The collected ovaries were washed three times in warm physiological saline supplemented with 5 mg/ml kanamycin (GIBCO-BRL Life Technologies, Merelbeke, Belgium). Subsequently, cumulus oocytes complexes were aspirated from 4 to 8 mm diameter follicles and cultured in groups of 60 in 500 μl maturation medium-containing TCM199 (Life Technologies, Ghent, Belgium) supplemented with 20% heat-inactivated fetal bovine serum (FBS) (Biochrom AG, Berlin, Germany) for 22 h at 38.5°C in 5% CO_2_ in the air. Frozen-thawed bovine spermatozoa from Holstein bulls were separated through a 45% and 90% Percoll gradient (GE healthcare Biosciences, Uppsala, Sweden). The final sperm concentration of 1 × 10^6^ spermatozoa/ml was adjusted in IVF-Tyrode’s albumin-lactate-pyruvate (IVF-TALP), consisting of bicarbonate-buffered Tyrode solution supplemented with 6 mg/ml and 25 μg/ml heparin bovine serum albumin (BSA) (Sigma, Schnelldorf, Germany). Matured oocytes were washed in 500 μl IVF-TALP medium and were incubated with spermatozoa. After incubation for 21 h, presumed zygotes were vortexed for 3 min to remove spermatozoa and cumulus cells, washed with IVF-TALP and transferred to 20 μl drops of synthetic oviductal fluid supplemented with ITS (5 μg/ml Insulin + 5 μg/ml Transferrin + 5 ng/ml Selenium) and 4 mg/ml BSA. Culture occurred individually in drops of 20 μl, covered with mineral oil at 38.5°C in 5% CO_2_, 5% O_2_ and 90% N_2_.

Bovine embryos were divided into groups according to the first cleavage patterns, as described previously (Dinnyes, 1999; Amarnath et al., 2007; Sugimura et al., 2017). Time points of first cleavage [24.2–33.8 h post insemination (hpi)] were listed up and were divided in quartiles. The first quartile was considered as “fast,” the second and third quartiles were considered as “intermediate” and the last quartile was considered as “slow.” More specifically, individual droplets were viewed microscopically at two time points (26.6 and 31.4 hpi), and three groups were produced according to the embryos’ cleavage pattern: “fast” (cleavage occurred before 26.6 hpi), “intermediate” (cleavage occurred between 26.6 and 31.4 hpi) and “slow” (cleavage had not occurred yet at 31.4 hpi). Additionally, the developmental competence of each embryo was microscopically viewed and assessed at 8 days post insemination (dpi), enabling a division into two subgroups (degenerate embryos and blastocysts). Eventually, the embryos were divided into six groups: FB (fast cleaving blastocyst), IB (intermediate cleaving blastocyst), SB (slow cleaving blastocyst), FD (fast cleaving degenerate), ID (intermediate cleaving degenerate), and SD (slow cleaving degenerate). Conditioned medium of single embryos was collected (17.5 μl each droplet) and pooled for each of the six groups.

### miRNA Extraction

At 8 dpi, the CM was collected and miRNA was extracted with the miRNeasy Serum/Plasma kit (Qiagen, Germantown, United States). To meet the miRNA-sequencing minimum concentration requirement, RNA was extracted from CM (three replicates of 3 mL each) and was concentrated with the RNeasy MinElute Cleanup kit (Qiagen, Germantown, United States). Finally, the quality and concentration of the RNA samples were examined using an RNA 6000 Pico Chip (Agilent Technologies, Carlsbad, CA, United States) and a Quant-iT RiboGreen RNA Assay kit (Life Technologies, Carlsbad, CA, United States), respectively. The total RNA isolated from CM ranged from 1.982 to 2.448 ng/μl. The FB and FD group were excluded because the required amount of secreted miRNAs from the IVF culture system for sequencing was not obtained.

### Small RNA Library Construction and Deep Sequencing

Small RNA library construction was performed with the Tailormix v2 kit (SeqMatic, Fremont, CA, United States). The quality-ensured RNA-seq libraries were pooled and sequencing was performed in triplicate on the Illumina Miseq (NxtGnt sequencing facility, Gent, Belgium).

### Small RNA-Sequencing Data Analysis and Differential Expression Analysis

Identification of known miRNAs, prediction of putative novel miRNAs and reading counting were done using the mirPRo pipeline (Shi et al., 2015). MicroRNA data from the miRBase (v21) (Griffiths-Jones et al., 2006) and the annotated cow genome (GCA_000003055.3) were used as reference. Differential expression between sample groups was statistically tested in R (Ihaka and Gentleman, 1996) with both EdgeR (Robinson et al., 2010) and DESeq2 (Love et al., 2014) via the SARTools wrapper (Varet et al., 2016). Two comparisons were made after RNA-Sequencing: IB vs. SB; (I + S) Degenerate vs. (I + S) Blastocysts. The results were considered statistically significant when the Benjamini-Hochberg corrected *p*-value was <0.05.

### Pathway Analysis

The functional analysis of the differentially expressed genes between the groups was performed using DAVID (Huang et al., 2008, 2009) (predicted target genes as input) and miRWalk (Dweep and Gretz, 2015) (miR-30c and miR-10b as input) in terms of enrichment of gene ontologies (GO). In addition, a pathway analysis was performed using the KEGG database to identify the significant pathways affected by the differentially expressed miRNAs. The Benjamini-Hochberg corrected *p*-values <0.05 were considered statistically significant.

### RT-qPCR

To verify the results of the miRNA sequencing, five mature miRNAs were quantified using RT-qPCR (real-time quantitative PCR). Accordingly, total RNA samples (including miRNAs) isolated from CM (three additional biological replicates of 200 μl each) were reverse transcribed using a miScript II RT kit (Qiagen, Germantown, MD, United States) and subsequently quantified with a miScript SYBR Green Kit containing 10 × miScript Universal Primer (Qiagen, Germantown, MD, United States). *U6* (Mondou et al., 2012; Abd El Naby et al., 2013) was quantified to normalize miRNA expression levels.

To check the intracellular expression of the differentially released miRNAs and if miR-30c is taken up by embryos, miRNAs were quantified using RT-qPCR. Total RNA samples (including miRNAs) isolated from embryos (three replicates of approximate 5 embryos each) using the miRNeasy Mini kit (Qiagen, Germantown, United States) and reverse transcribed using a miScript II RT and subsequently quantified with a miScript SYBR Green Kit containing 10 × miScript Universal. *U6* was quantified to normalize miRNA expression levels.

Additionally, embryos and MDBKs were used to analyze mRNA abundance of *CDK12* and DDR genes. Total RNA samples were isolated from embryos (three replicates of approximate 5 embryos each) and MDBKs using the RNeasy Micro kit (Qiagen, Germantown, MD, United States) and reverse transcribed using the iScript cDNA synthesis kit (BioRad, Brussels, Belgium). The mRNA levels were quantified with a SsoAdvanced Universal SYBR Green Supermix kit (BioRad, Brussels, Belgium). *GAPDH* (Herrmann et al., 2013; Li et al., 2016), which has been proved to be a stable reference gene in our sample (data not shown), was quantified to normalize mRNA expression levels.

All reactions were performed in triplicate, and the 2^-ΔΔCt^ method was used to analyze the data. The primer sequences used for RT-qPCR are listed in Supplementary Table S1.

### miR-30c Mimics Supplementation to Embryos Culture Medium

Since individually cultured embryos have less tolerance when compared to group cultured embryos (Goovaerts et al., 2009; Wydooghe et al., 2014a,b) and they easily die after changing the culture environment, group culture was performed for miR-30c functional analysis instead of individual culture. The IVF embryos were produced according to the previously described protocol. This time, however, presumed zygotes were vortexed for 3 min after 21 h incubation, washed with IVF-TALP and transferred to drops of SOF supplemented with ITS, BSA and miR-30c mimics (chemically synthesized, double-stranded RNAs which mimic mature endogenous miRNAs after delivery to cells) or control mimics (chemically synthesized, double-stranded RNAs which have no homology to any known microRNA or mRNA sequences) (Qiagen, Germantown, United States) with a final concentration of 1 μM according to the instructions. Culture occurred in groups of 25 in drops of 50 μl, covered with mineral oil at 38.5°C in 5% CO_2_, 5% O_2_, and 90% N_2_. On 8 dpi, blastocyst rates were calculated. Blastocysts were collected for RT-qPCR or assessed with apoptosis staining.

### TUNEL Staining and Differential Apoptotic Staining

TUNEL staining was performed using a previously described protocol (Ortiz-Escribano et al., 2017) with an *in situ* cell death detection kit (Sigma, St. Louis, MO, United States). Briefly, ∼20 blastocysts for each group were collected and fixed in 4% paraformaldehyde at room temperature (RT) for 1 h, and then permeabilized in 0.1% Triton X-100 at RT for 10 min. Afterward, blastocysts were stained with 20 μl TUNEL mixture for 1 h at 37C and subsequently stained with 10 μg/ml DAPI for 10 min. The embryos were mounted on the slides and were examined using a 20× water immersion objective on a Leica TCS-SP8 X confocal microscope (Leica microsystems, Wetzlar, Germany). The apoptosis ratio was expressed as the total number of TUNEL-positive cells relative to the total number of the cells per blastocyst.

Differential apoptotic staining was performed using previously described protocols (Wydooghe et al., 2011; Lu et al., 2019). The first day, ∼20 blastocysts for each group were fixed in 4% paraformaldehyde for 1 h and put in a 4-well dish in permeabilization solution (0.5% Triton X-100 + 0.05% Tween) in phosphate buffered saline (PBS) at RT for 1 h. After washing the blastocysts 3 times during 2 min in PBS-BSA, they were incubated in 2N HCl at RT for 20 min and then in 100 mM Tris–HCl at RT for 10 min. The blastocysts were washed (3 times during 2 min) and then put into 500 μl of blocking solution at 4C overnight. The second day, the blastocysts were washed again (3 times during 2 min) and incubated in primary CDX-2 antibody (Biogenex, San Ramon, United States) at 4C overnight. On the third day, the blastocysts were washed twice for 15 min and subsequently incubated in blocking solution containing the rabbit active caspase-3 antibody (Cell Signaling Technology, Leiden, Netherlands) overnight at 4C. On day four, the blastocysts were incubated in blocking solution containing the goat anti-mouse Texas Red antibody at RT for 1 h and were subsequently incubated in blocking solution containing the goat anti-rabbit FITC antibody at RT for 1 h. The blastocysts were washed twice for 15 min and incubated at RT for 20 min in a dilution 1: 200 Hoechst in PBS-BSA in the dark. All slides were examined using a 63 × water immersion objective on a Leica TCS-SP8 X confocal microscope. The apoptosis ratio was expressed as the total number of Caspase-3-positive cells relative to the total number of the cells per blastocyst.

### Plasmid Construction

The full-length coding sequence of *CDK12* (4473 bp) (NM_001205701.1) was amplified from MDBK cDNA and was inserted into a pEGFP-N1 vector via NheI and XhoI sites for construction of the *CDK12-*overexpressing vector. The empty vector (mock) was used as a negative control. The *CDK12* 3′-UTR (282 bp) containing the predicted miR-30c binding site was amplified from bovine genomic DNA and inserted into a psi-CHECK2 vector (Promega, Madison, United States) via NotI and XhoI sites and confirmed by sequencing. To test whether the predicted miR-30c target site in the *CDK12* 3′-UTR is critical for the miR-30c-mediated repression of *CDK12* expression, the seed sequence of the predicted miR-30c’s binding site was changed (Wu et al., 2017; Figure 4A). Primers for vector construction are listed in Supplementary Table S1.

### Dual-Luciferase Reporter Assay

The miR-30c mimics/control mimics and luciferase reporter plasmids were co-transfected into HEK293T cells using Lipofectamine 2000 (Invitrogen, Carlsbad, United States). After 24 h of transfection, the Renilla and Firefly luciferase were assayed using the Dual Luciferase Reporter Kit (Promega, Madison, WI, United States).

### Cell Culture and Transfection

The HEK293T cells and MDBK cells were cultured at 37C in 5% CO_2_ in DMEM media (Thermo Fisher Scientific, Waltham, MA, United States) supplemented with 10% FBS (VWR, Radnor, United States), 100 U/ml penicillin and 100 mg/ml streptomycin. miR-30c mimics/inhibitor and their negative controls were delivered into MDBK cells using Hiperfect reagent (Qiagen, Germantown, MD, United States) following the manufacturer’s instructions. The short-interfering RNA (siRNA) targeting *CDK12* and a non-target control siRNA (si-NTC) were purchased from Qiagen (Germantown, MD, United States). SiRNA or the overexpressing vector was transfected into MDBK cells using Lipofectamine 2000 according to the manufacturer’s instructions. Protein or total RNA were extracted for western blotting (WB) or RT-qPCR 48 or 24 h after transfection.

### Western Blotting

Cells were collected after 48 h of transfection and lysed using Radioimmunoprecipitation lysis buffer consisting of 50 mM Tris–HCl (pH 7.5), 150 mM NaCl, 0.1% SDS, 1% NP-40, 0.5% sodium deoxycholate and protease inhibitors. The samples were denatured at 100C for 10 min before loading onto 10% SDS-polyacrylamide gels. Separated proteins were then transferred onto nitrocellulose membranes and blocked with 5% non-fat milk in PBS with 0.1% Tween-20 for overnight. Membranes were then incubated overnight with 1/1000 rabbit anti-*CDK12* (Novus Biologicals, Abingdon, United Kingdom) and 1/1000 rabbit anti-β-actin (Novus Biologicals, Abingdon, United Kingdom). After three washes, the membranes were incubated with HRP-conjugated goat anti-rabbit IgG (H + L) (Novus Biologicals, Abingdon, United Kingdom) for 2 h at room temperature. Signals were revealed by autograph using SuperSignal West Femto Maximum Sensitivity Substrate (Thermo Fisher Scientific, Waltham, United States).

### Cell Cycle Assays: PI Staining and Flow Cytometry

Madin-Darby bovine kidney cells were cultured in 6-well plates for 48 h after transfection and were stained with propidium iodide (PI) at a final concentration of 50 μg/ml PI and 100 μg/ml RNase A in PBS. Then, the cells were analyzed using Accuri^TM^ C6 flow cytometry (BD, Erembodegem, Belgium) collecting 50000 events. All experiments were replicated three times.

### Cell Proliferation Assays: WST-1 Colorimetric Assay

WST-1(4-(3-(4-iodophenyl)-2-(4-nitrophenyl)-2H-5-tetrazolio)-1,3-benzene disulfonate) (Merck, Kenilworth, United States) was used for cell proliferation analysis. The assay was performed using 96-well plates with ∼20000 cells. After 48 h of transfection, 10 μl of WST-1 was added to 90 μl samples. The samples were measured at 450 nm wavelength (570 nm as a reference wavelength) using an EZ read 400 microplate reader (Biochrom, Holliston, United States). Cell viability was then calculated by comparing the absorbance values of sample groups after background subtraction. All experiments were replicated three times.

### Statistical Analysis

The data are presented as mean ± S.D and derived from at least three independent experiments. The statistical analyses were performed using ANOVA followed by Tukey’s test or Student’s *t* test. For each analysis, *P* < 0.05 was considered significant.

## Results

### Intermediate Cleaving Embryos Result in a Higher Blastocyst Rate Compared to Slow Cleaving Embryos

**FIGURE 1:**
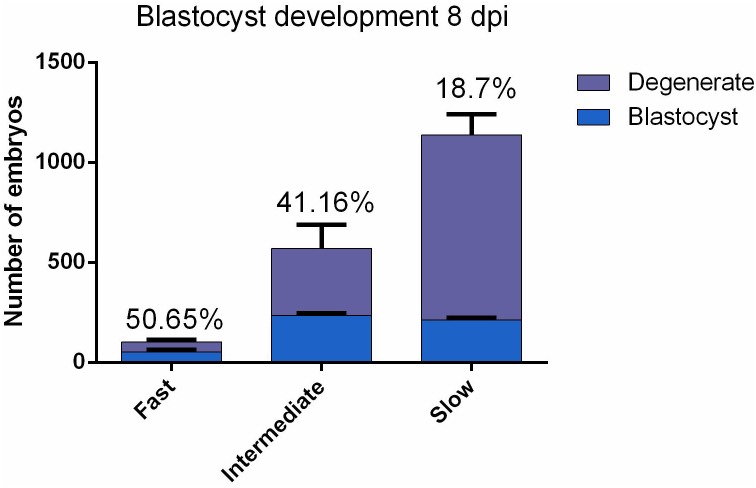
Bovine embryos (*n* = 1808 for each replicate) were individually cultured and were grouped according to the two-cell stage cleavage pattern: fast, intermediate and slow. The developmental competence of each embryo was assessed at 8 dpi, enabling a division into two subgroups: degenerate and blastocyst. Data are presented as mean ± SD of three experiments.

According to the timing of the first cell division, 1808 individually cultured embryos for each of three replicate were labeled as either fast, intermediate or slow cleaving and evaluated at 8 dpi for developmental competence. Intermediate embryos produced significantly (*P* = 0.027) more blastocysts in comparison to the slow embryos (41.16 and 18.7%, respectively; Figure 1). No statistically distinctive differences (*P* = 0.24) were found between fast and intermediate cleaving embryos (50.65 and 41.16%, respectively; Figure 1). The fast group was excluded for sequencing because not enough RNA was obtained due to the low number of embryos belonging to this group.

### miRNAs Secreted by Bovine Embryos

**FIGURE 2:**
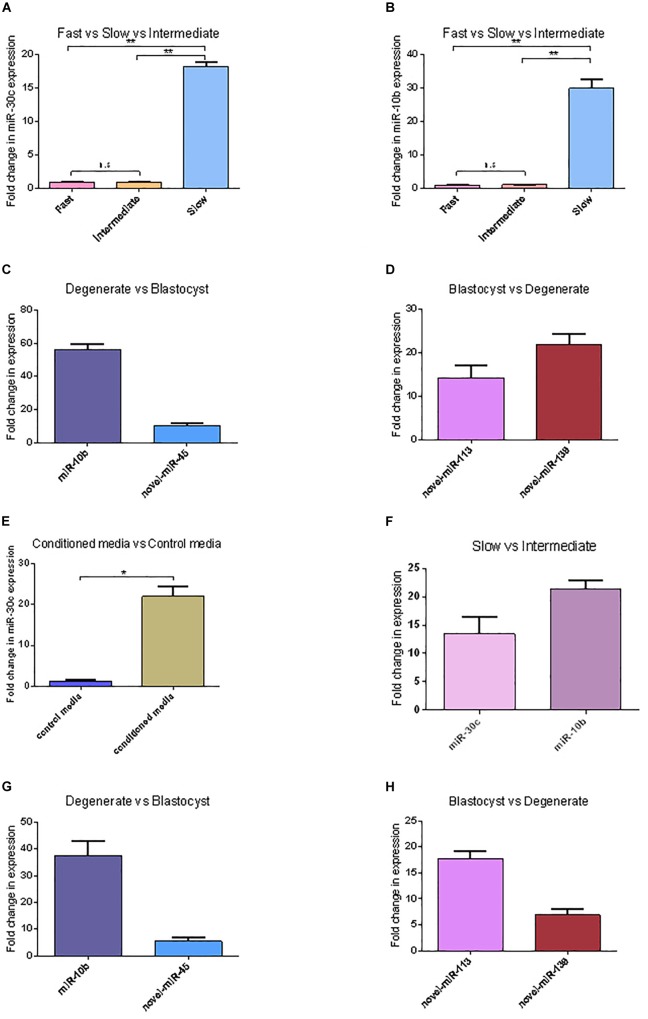
miRNAs’ differential expression between embryos with different cleavage patterns and different blastocyst formation. **(A,B)** The relative expression levels of miR-30c and miR-10b between the CM of fast, intermediate and slow developing embryos were detected using RT-qPCR. **(C,D)** Relative expression of miRNAs between CM of degenerate embryos and blastocyst. **(E)** Relative expression of miR-30c between conditioned media and control media. **(F)** Relative expression of miR-30c and miR-10b between the intermediate and slow developing embryos. **(G,H)** Relative expression of miRNAs between CM of degenerate embryos and blastocyst. Data are presented as mean ± SD of three experiments. (^∗^*P* < 0.05, ^∗∗^*P* < 0.01, n.s, no significance).

In total 294 miRNAs were found in conditioned media (CM) after sequencing (MicroRNAs sequencing data are available in the GEO database under the accession number PRJNA492220): 114 known miRNAs and 180 potential novel miRNAs. The uncorrected *p*-value was indicative of differential secretion from embryos with different cleavage patterns and different development competences for the following miRNAs: miR-30c and miR-10b were secreted more in slow cleaving embryos’ CM compared with the CM of intermediate cleaving embryos; miR-10b, miR-novel-44, and miR-novel-45 were more abundant in CM from degenerate embryos than in that of blastocysts, while miR-novel-113 and miR-novel-139 were more abundant in blastocyst’s CM than degenerate’s CM (Table 1). However, with the low sample size, due to the practical difficulty to obtain enough CM, it was unsurprising that none of the differences remained significant after multiple testing with the Benjamini-Hochberg corrected *p*-value. Consequently, the sequencing results of 5 of the 6 above mentioned miRNAs were confirmed using RT-qPCR (novel-miR-44 has the same mature sequence as novel-miR-45) (Figure 2). RT-qPCR showed that miR-30c and miR-10b have an 18 (*P* = 0.00072) and 30 (*P* = 0.00017) fold higher expression in the CM from slow cleaving embryos in comparison to intermediate cleaving embryos (Figure 2A,B). The expression levels of both these two miRNAs in the CM of fast cleaving embryos and intermediate cleaving embryos showed no significant difference (Figure 2A,B). MiR-10b and novel-miR-45 showed a 55 (*P* = 0.00000) and 8 (*P* = 0.0068) fold higher expression in the CM from degenerate embryos compared to blastocysts (Figure 2C). Novel-miR-113 and novel-miR-139 displayed, respectively 14 (*P* = 0.0027) and 22 (*P* = 0.00033) fold higher expression in the CM of blastocysts than in that of degenerate embryos (Figure 2D). In addition, miR-30c was found to be 20 (*P* = 0.00067) times more abundant in CM compared to control media (Figure 2E).

**Table 1:** The differentially expressed miRNAs (*p* < 0.05) in CM content from individually cultured bovine embryos (I, intermediate cleaving; S, slow cleaving).

(I + S) Degenerate embryos vs. (I + S) Blastocysts			
	
Name	Fold Change	*p*-value	Benjamini-Hochberg corrected *p*-value
bta-miR-10b	3.047	0.006246833	0.224885994
bta-novel-miR-113	0.268	0.012946778	0.233041999
bta-novel-miR-45	3.934	0.031555548	0.255968877
bta-novel-miR-44	3.969	0.034171109	0.255968877
bta-novel-miR-139	0.124	0.035551233	0.255968877
**SB vs. IB**		
bta-miR-30c	17.857	0.041729634	0.707989068
bta-miR-10b	3.831	0.048368042	0.707989068


The intracellular miRNAs expression was also validated using RT-qPCR and similar results were obtained. miR-30c and miR-10b have a 13 (*P* = 0.0031) and 21 (*P* = 0.00044) times higher expression in slow cleaving embryos in comparison to intermediate cleaving embryos (Figure 2F). MiR-10b and novel-miR-45 show a 37 (*P* = 0.0004) and 5 (*P* = 0.0081) times higher expression in degenerate embryos compared to blastocysts (Figure 2G). Novel-miR-113 and novel-miR-139 displayed 18 (*P* = 0.00091) and 7 (*P* = 0.0062) times higher expression in blastocysts than degenerate embryos (Figure 2H).

### Pathway Analysis

**FIGURE 3:**
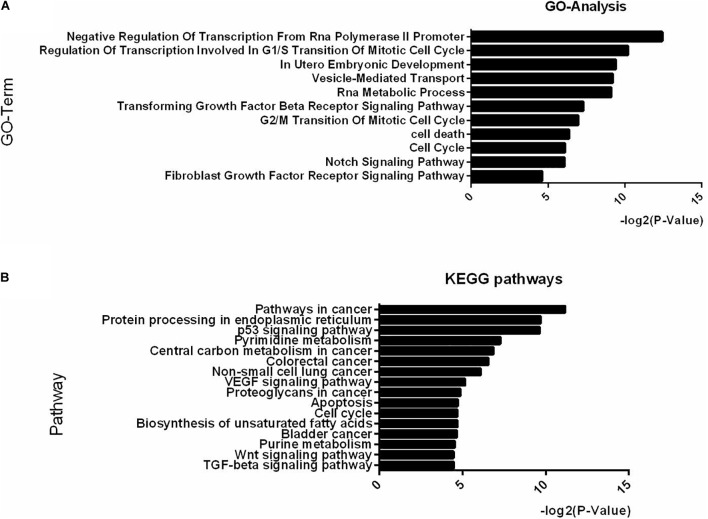
Top-ranking GO terms and KEGG pathways enriched in genes differentially expressed in IB vs. SB. **(A,B)** Enriched GO and KEGG pathways with Benjamini-Hochberg corrected *p*-value <0.05.

Examination of the GO analysis results of the differentially expressed miRNAs between IB and SB revealed that 11 biological processes, among which “in utero embryonic development,” “cell cycle,” “fibroblast growth factor receptor signaling pathway,” and “Notch signaling pathway” were over-represented (Figure 3A). Additionally, 16 KEGG pathways, with as top hits: the p53 signaling pathway, the Wnt signaling pathway, the TGF-beta signaling pathway and apoptosis were over-represented (Figure 3B). These GO-terms and pathways enriched with targets provide an intriguing clue to the biological consequences of miRNAs differential secretion from embryos with different cleavage patterns.

### miR-30c Mimics Can Be Taken Up by Bovine Embryos and Increase Embryo Apoptosis

**FIGURE 4:**
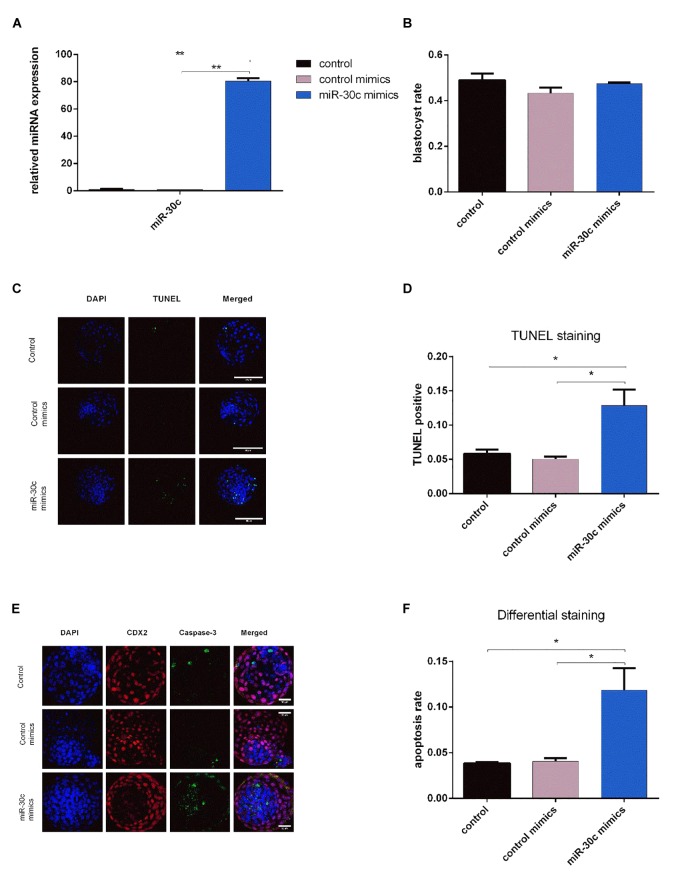
Effects of miR-30c mimics on miR-30c expression in embryos, embryo growth and apoptosis **(A)** Embryos were treated with miR-30c mimics or control mimics and miR-30c expression was evaluated using RT-qPCR. The blastocyst rate was assessed at 8 dpi **(B)** and cell apoptosis was determined by TUNEL staining **(C,D)** and differential apoptotic staining **(E,F).** Data are presented as mean ± SD of three experiments. (^∗^*P* < 0.05, ^∗∗^*P* < 0.01).

miR-30c has been shown to regulate cell cycle and proliferation in human breast cancer cells, glioma cells, hematopoietic cells, osteoblast cells and mice embryonic carcinoma cells (Li et al., 2012; Quintavalle et al., 2013; Shukla et al., 2015; Liu et al., 2016), thus, combining the above sequencing/RT-qPCR results with information from the literature we hypothesized that miR-30c can be taken up by embryos and might influence embryonic development through regulation of the cell cycle. To test this hypothesis, we added the miR-30c mimics into the IVF culture medium at 21 hpi, thus allowing miR-30c mimics to influence embryos for at least 5 to 10 h before they reach the 2-cell stage (26–31 hpi). RT-qPCR results showed that the miR-30c levels were approximate 80 times higher in miR-30c mimics treated embryos compared to the control mimics group (Figure 4A), indicating that miR-30c was taken up by the embryos.

No significant difference was found in blastocyst rate between miR-30c mimics group and control mimics group (Figure 4B). However, TUNEL staining showed that the miR-30c mimics group had an apoptosis rate of 12.86% whereas that of the control mimics group was 5.05% (Figure 4C,D). Similarly, differential apoptotic staining showed that the miR-30c mimics group had an apoptosis rate of 11.85% whereas that of the control mimics group was 4.05% (Figure 4E,F).

### miR-30c Directly Targets Cell Progression Regulator *CDK12*

**FIGURE 5:**
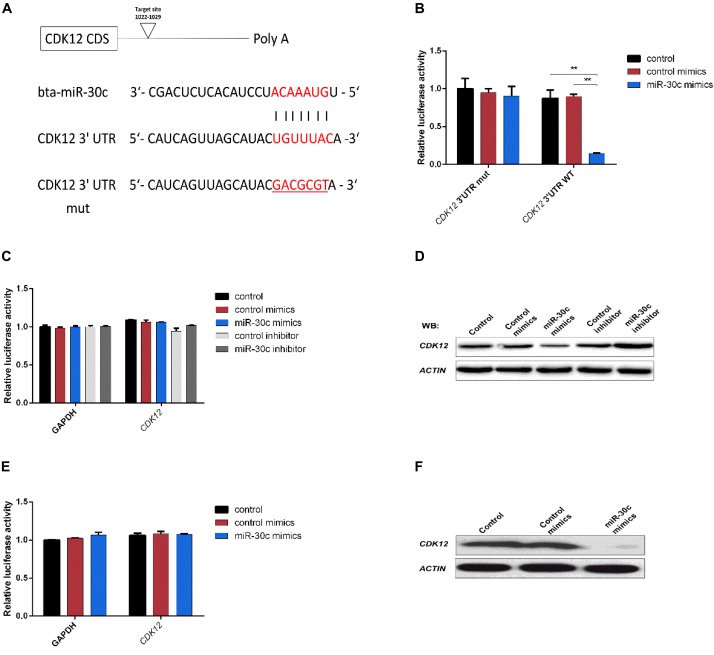
*CDK12* is a direct target of miR-30c. **(A)** 3′-UTR analysis of *CDK12* containing putative regions that match the seed sequence of miR-30c. The mutated nucleotides are underlined. **(B)** Overexpression of miR-30c inhibited the Renilla luciferase activities. HEK-293T cells were cotransfected with 5 nM miR-30c mimics and 500 ng of reporter plasmid containing the WT or MUT-type UTRs. 24 h later, Renilla luciferase values were normalized against firefly luciferase and presented. **(C,D)** miR-30c mimics or inhibitor were transfected into MDBKs. After 48 or 24 h, cells were harvested for western blot or RT-qPCR. **(E,F)** Embryos were treated with miR-30c mimics or control mimics and the relative levels of *CDK12* was detected using RT-qPCR and WB. Data are presented as mean ± SD of three experiments. (^∗∗^*P* < 0.01).

Different miRNA target prediction methods may produce different results, thus we adopted the method from Ozen (Ozen et al., 2007) and Li (Li et al., 2011). If a target was identified by at least three of six used different algorithms (TargetScan, miRDB, PicTar, miRanda, miRWalk and Tarbase), it was considered likely to be a miRNA target. Of the putative target genes identified in this way, *CDK12* (identified by Targetscan, miRDB and miRanda) was chosen for further analysis. This gene was previously shown to be required for the prevention of apoptosis (Bartkowiak et al., 2015; Juan et al., 2016) and to protect cells from genomic instability and inhibit cell differentiation (Blazek et al., 2011; Dai et al., 2012) through the regulation of DDR genes in human and mouse. The 3′-UTR segment of the bovine *CDK12* gene containing the putative miR-30c target binding site region (Figure 5A) was amplified and cloned into luciferase reporter vector psi-CHECK2 and subsequently transfected to HEK293T cells. As shown in Figure 5B, the miR-30c mimics dramatically suppressed the activities of wild-type (WT) 3′-UTRs of *CDK12*, while the mutated 3′-UTR binding site (MUT) was unaffected. To further confirm the regulatory relationship between miR-30c and *CDK12*, RT-qPCR, and WB were performed to determine the *CDK12* mRNA and protein levels in MDBKs. The results showed that *CDK12* was suppressed by miR-30c mimics and enhanced by miR-30c inhibitors at the protein level (Figure 5C) rather than the mRNA level (Figure 5D). The direct target relationship was also analyzed in embryos: miR-30c mimics were supplemented into embryos culture medium and then *CDK12* expression was evaluated using RT-qPCR and WB. Not surprisingly, embryos showed the similar results as MDBKs (Figure 5E,F). Collectively, these results show that miR-30c directly targets *CDK12* and inhibits its translation instead of degrading mRNA.

### miR-30c Overexpression and *CDK12* Downregulation Direct Transcription of Key DDR Genes

**FIGURE 6:**
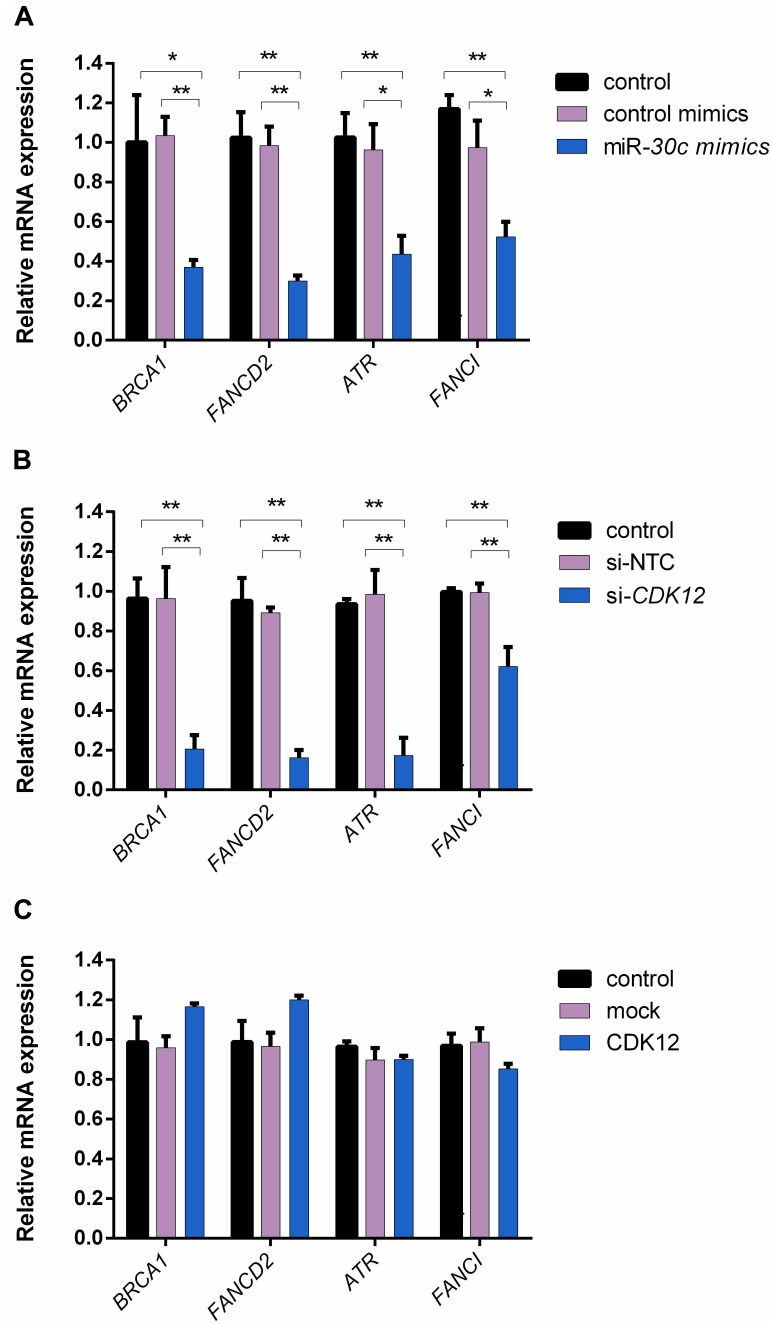
The delivery of miR-30c mimics into embryos and *CDK12* downregulation in MDBKs regulate the expression of DDR genes. **(A)** Embryos were supplemented with miR-30c mimics or control mimics. The blastocysts (8 dpi) were then subjected to RT-qPCR. **(B,C)** MDBKs were transfected with siRNAs or the overexpressing vector for 24 h. The cells were then subjected to RT-qPCR. Data are presented as mean ± SD of three experiments. (^∗^*P* < 0.05, ^∗∗^*P* < 0.01).

Given that *CDK12* is involved in DNA repair (Paculová et al., 2017) and has been proven to be a target gene inhibited by miR-30c in our study, we hypothesized that miR-30c may suppress cell cycle progression by inhibiting DDR pathways. A previous study on mouse embryos showed that four DDR genes, namely *Brca1*, *Fancd2*, *Fanci*, and *Atr*, had a reduced expression in the absence of *CDK12* (Juan et al., 2016). To our knowledge, in bovine, the relationship among miR-30c, *CDK12*, and DDR pathway has not been investigated yet. Here we examined the expression of these four genes using RT-qPCR after supplementing miR-30c mimics into embryos culture medium and modulating *CDK12* expression in MDBKs. As shown in Figure 6A, the delivery of miR-30c significantly decreased mRNA levels of all four investigated DDR genes *BRCA1*, *FANCD2*, *FANCI*, and *ATR* in embryos. As shown in Figure 6B, downregulation of *CDK12* also significantly decreased mRNA levels of the above four genes. We also examined the expression of DDR genes after overexpressing *CDK12* using the previously mentioned vector construct. As shown in Figure 6C, overexpression of *CDK12* did not have a significant effect on the mRNA level of these DDR genes.

### miR-30c Suppresses the Cell Cycle, While *CDK12* Promotes the Cell Cycle

**FIGURE 7:**
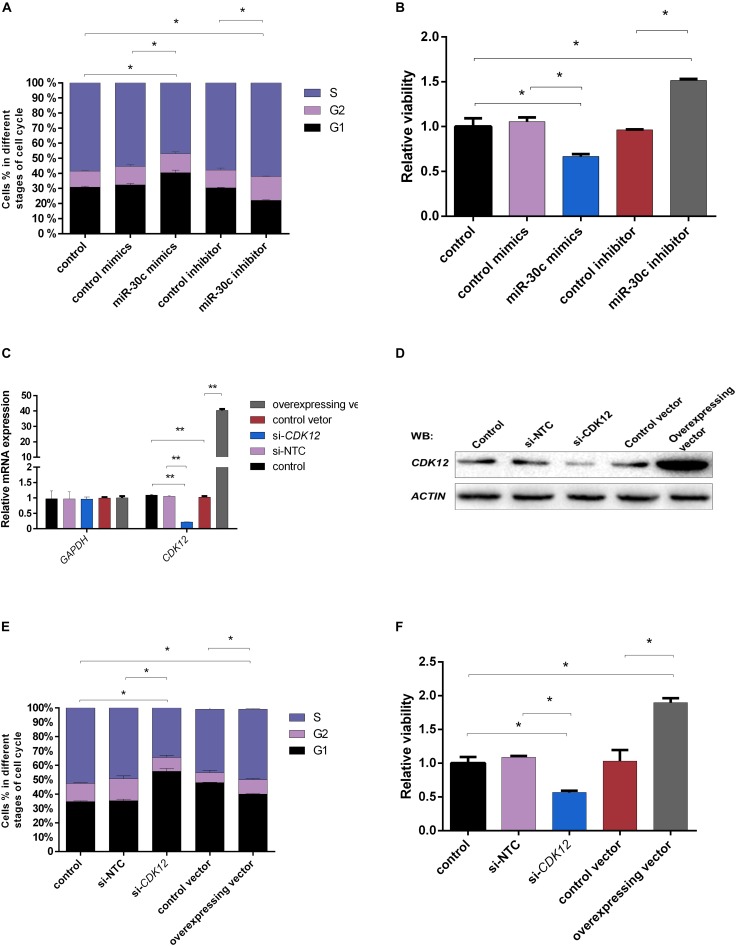
Effects of miR-30c mimics/inhibitor and *CDK12* overexpression/downregulation on cell process. **(A,B)** MDBK cells were reverse transfected with miR-30c mimics or inhibitor for 48 h. The cells were then subjected to cell cycle assay by PI staining and cell proliferation assay by WST-1 assay. **(C–F)** MDBK cells were reverse transfected with siRNAs or overexpressed vector for 48 h. The cells were then subjected to RT-qPCR, WB, cell cycle assay by PI staining and cell proliferation assay by WST-1 assay. Data are presented as mean ± SD of three experiments. (^∗^*P* < 0.05, ^∗∗^*P* < 0.01).

Although miR-30c has been shown to regulate cell progression in human and mouse (Quintavalle et al., 2013; Liu et al., 2016), this regulatory relationship is still unclear in bovine cells. Considering the fact that the compaction of embryos makes it difficult to use them for flow cytometry analysis, further studies were performed using the bovine cell line MDBKs. PI staining was used to determine the effect of miR-30c mimics or inhibitors on the MDBK cell cycle. As shown in Figure 7A, cell cycle phase distribution determined by flow cytometry displayed 8% increase of treated cells in the G1 phase after delivery of miR-30c mimics, indicating the cell growth suppression, while delivery of miR-30c inhibitors resulted in an 8% decrease of cells in G1 phase.

*CDK12* expression was assessed after siRNA or vector transfection. RT-qPCR (Figure 7C) and WB (Figure 7D) showed that *CDK12* expression was indeed upregulated by the vector construct and downregulated by siRNA at transcriptional level, showing their usefulness for the next experiments. Transfection of the *CDK12* overexpressing construct resulted in an 8% decrease of cells in the G1 phase compared with the control group, whereas knockdown of *CDK12* using siRNA resulted in a 20% increase of cells in the G1 phase and a 15% decrease in the S phase compared with si-NTC (Figure 7E).

### miR-30c Decreases Cell Viability, While *CDK12* Increases Cell Viability

The MDBKs cellular metabolic activity, indicative of the cell proliferation, was monitored after addition of miR-30c mimics or inhibitors using the WST-1 assay. As shown in Figure 7B, miR-30c mimics led to a significant decrease in cell viability (37%), while miR-30c inhibitors increased cell viability (57%).

As shown in Figure 7F, *CDK12* overexpression increased cell viability (84%), while *CDK12* inhibition led to a 49% decrease in cell viability.

## Discussion

Timing of cleavage is regarded as an important marker to assess embryo quality (Gutierrez-Adan et al., 2015) and it has been shown that rapid cleaving embryos are of better quality than slower cleaving embryos (Meirelles et al., 2004; Vandaele et al., 2006). Because it has been demonstrated that an embryo’s potential is determined more in the early developmental stages than in later developmental stages (Wong et al., 2010; Milewski and Ajduk, 2017; Milewski et al., 2018), we chose the 2-cell stage to assess the samples regarding evaluation of embryos quality, instead of the 4-cell stage or the morula stage. In our study, fast and intermediate cleaving embryos produced significantly more blastocysts compared to the slow cleaving embryos (50.65 and 41.16% vs. 18.7%), confirming the above theory.

In addition to their intracellular function, secreted miRNAs may play a significant role in intercellular communications (Vickers et al., 2011; Boon and Vickers, 2013; Yang et al., 2018). However, the dynamics of miRNA secretion and their transfer mechanisms are still poorly understood. Secreted miRNAs have been found to be related to cell growth, invasion, migration, dissemination as well as metastasis and impairment of the immune system response (Schwarzenbach et al., 2014). Furthermore, they have potential as biomarkers for cancer and benign diseases, thus raising the questions whether and how secreted miRNAs influence embryo development and if they can be used as non-invasive biomarkers for embryo quality. Given the current methods for miRNA detection, the main limitation is the low abundance of miRNAs in CM. However, miRNAs secreted by a single human embryo have been successfully detected and extracted (Capalbo et al., 2016), indicating the potential application for bovine embryos. In our study, to obtain a sufficient amount of miRNAs for sequencing, we concentrated the CM from 167 embryos for each replicate and thus achieved at least 1 million raw reads. The potential of secreted miRNAs as biomarkers relies mainly on their high stability and their capacity to reflect embryo developmental status and their prognostic abilities in relation to IVF success and pregnancy outcome. Although there are several recent studies focusing on miRNAs in culture media (Rosenbluth et al., 2014; Kropp and Khatib, 2015) and body fluids, such as follicular fluid (Sohel et al., 2013) and endometrium (Vilella et al., 2015), providing an indication of the developmental competence of embryos, improvements in detection techniques and more knowledge of the miRNA signaling is needed in order to use secreted miRNA as biomarkers in embryonic development. In addition, not only technical aspects currently limit the use of secreted miRNAs as biomarkers in culture media and also in other body fluids; to date the source of secreted miRNAs is not clear. Therefore, more extensive studies are necessary to clarify whether secreted miRNAs detected in extracellular environment are the product of dead cells or are secreted in a tissue-specific manner. Furthermore, studies with large samples sizes are needed and some aspects of experimental reliability must be assessed before secreted miRNAs can be used as biomarkers.

Apart from the easy detection, a biomarker should be clearly discriminatory for the state to be defined, in casu the developmental competence of the embryo. Here, we demonstrated that miRNAs are differentially secreted from bovine embryos with different cleavage patterns and different qualities: miR-30c and miR-10b were differentially expressed between slow and intermediate cleaving embryos’ CM; miR-10b, miR-novel-113, miR-novel-44, miR-novel-45, and miR-novel-139 were differentially expressed between blastocyst’s and degenerate’s CM.

Among the differentially expressed miRNAs, miR-30c was found to be 18 times more abundant in slow cleaving embryos’ CM vs. intermediate cleaving embryos’ CM. This distinct difference makes it a suitable biomarker candidate for the developmental capacity of bovine early embryos. To gauge the effect of miR-30c uptake by bovine embryos in correlation with the cleavage pattern and the proposed roles of miR-30c in cell proliferation in mouse (Liu et al., 2016), cell apoptosis in human and mouse (Li et al., 2010; Quintavalle et al., 2013; Liu et al., 2016), cell differentiation in human and mouse (Karbiener et al., 2011; Wu et al., 2012) and cell damage in human (Li et al., 2012), apoptosis assays were performed. RT-qPCR results confirmed that miR-30c was indeed taken up by bovine embryos and they showed a higher apoptosis rate, which is in agreement with previous findings that miRNAs could be both released and taken up by embryos (Kropp and Khatib, 2015; Vilella et al., 2015; Gross et al., 2017). The effect was further investigated using the bovine cell line MDBK. The delivery of miR-30c mimics to the MDBKs led to reduced cell proliferation and an arrest at G1 stage, while the delivery of miR-30c inhibitors resulted in the opposite effects, as expected. Previous studies on human embryos suggested that miR-30c can serve as a potential marker of blastocyst implantation potential (Capalbo et al., 2016; Noli et al., 2016). This is not surprising because although miR-30c is highly conserved between different species, it has been shown to act differently among different species. For instance, miR-30c was found to increase cell proliferation in mouse embryonal carcinoma cells (Liu et al., 2016), while it was also found to be a tumor suppressor miRNA in human cancers (Poudel et al., 2013; Shukla et al., 2015).

We also demonstrated for the first time that miR-30c downregulates *CDK12* expression at a post-transcriptional level both on bovine embryos and MDBKs. *CDK12* is a transcription-associated CDK that exerts control over Pol II-mediated transcription (Ekumi et al., 2015) and is essential for splicing and differentiation (Chilà et al., 2016). Intriguingly, recent research has shown that *CDK12* is essential for embryonic development and the maintenance of genomic stability by regulating the expression of DDR genes, and reduced expression of some of these DDR genes will subsequently trigger apoptosis (Juan et al., 2016; Chen et al., 2017). During early embryonic development, DNA replication is prominent and highly efficient DNA repair is crucial for proper embryo development. For instance, *Atr*- and *Brca1*-lacking embryos were reported to display growth retardation in mice (Liu, 1996; Brown and Baltimore, 2000). In our study, both the supplementation of miR-30c mimics into bovine embryos culture medium and the *CDK12* knockdown in MDBKs caused a decreased expression level of key DDR genes *BRCA1*, *FANCD2*, *FANCI*, and *ATR*. These results present evidence that miR-30c overexpression or *CDK12* downregulation reduces the expression of these DDR genes at the transcriptional level, leading to a potential failure of DNA damage repair. Interestingly, while *CDK12* overexpression increased cell cycle progression and cell proliferation, it had no effect on the mRNA level of those key DDR genes. This indicates that *CDK12* overexpression might influence cell cycle progression at other levels or through other mechanisms. For instance, in breast cancer cells, *CDK12* overexpression led to altered alternative last exon splicing of a subset of genes (Tien et al., 2017) and increased the invasiveness of a breast cancer cell line by decreasing the expression of the long isoform of DNAJB6 (Paculová and Kohoutek, 2017). A potential weakness of our study is that due to technical difficulties, part of the functional analysis of *CDK12* was done on MDBKs. It would be better if we can validate this mechanism in bovine embryos.

In summary, we have found 114 known miRNAs and 180 potential novel miRNAs in CM of bovine embryos. We have also identified miR-30c, which can be secreted and taken up by bovine embryos, as a novel potential biomarker related to bovine embryo apoptosis and reduced development. As miR-30c directly targets *CDK12* and downregulates DDR genes, it may exert its effects on cell cycle progression by inhibiting the DDR pathways.

## Author Contributions

XL performed the experiments and wrote the manuscript. YG provided the bioinformatics analysis for miRNA sequencing. EB contributed the qPCR experiments. KS was responsible for the embryo staining. KP helped to produce embryos. SMC, JC, PS, FVN, DD, AVS, and LP participated in the study design. All authors reviewed the manuscript.

## Conflict of Interest Statement

The authors declare that the research was conducted in the absence of any commercial or financial relationships that could be construed as a potential conflict of interest.

## References

[B1] Abd El Naby W. S. Hagos T. H. Hossain M. M. Salilew-Wondim D. Gad A. Y. Rings F. (2013). Expression analysis of regulatory microRNAs in bovine cumulus oocyte complex and preimplantation embryos. *Zygote* 21 31–51. 10.1017/S0967199411000566 22008281

[B2] Amarnath D. Kato Y. Tsunoda Y. (2007) Effect of the timing of first cleavage on in vitro developmental potential of nuclear-transferred bovine oocytes receiving cumulus and fibroblast cells. *J. Reprod. Dev.* 53 491–497. 10.1262/jrd.18112 17310082

[B3] Bartkowiak B. Liu P. Phatnani H. P. Fuda N. J. Cooper J. J. Price D. H. (2010). CDK12 is a transcription elongation-associated CTD kinase, the metazoan ortholog of yeast Ctk1. *Genes Dev.* 24 2303–2316. 10.1101/gad.1968210 20952539 PMC2956209

[B4] Bartkowiak B. Yan C. Greenleaf A. L. (2015). Engineering an analog-sensitive CDK12 cell line using CRISPR/Cas. *Biochim. Biophys. Acta* 1849 1179–1187. 10.1016/j.bbagrm.2015.07.010 26189575 PMC4556607

[B5] Blazek D. Kohoutek J. Bartholomeeusen K. Johansen E. Hulinkova P. Luo Z. (2011). The Cyclin K/Cdk12 complex maintains genomic stability via regulation of expression of DNA damage response genes, *Genes Dev.* 25 2158–2172. 10.1101/gad.16962311 22012619 PMC3205586

[B6] Boon R. A. Vickers K. C. (2013). Intercellular Transport of MicroRNAs. *Arterioscler. Thromb. Vasc. Biol.* 33 186–192. 10.1161/ATVBAHA.112.300139 23325475 PMC3580056

[B7] Brown E. J. Baltimore D. (2000). ATR disruption leads to chromosomal fragmentation and early embryonic lethality. *Genes Dev.* 14 397–402. 10691732 PMC316378

[B8] Capalbo A. Ubaldi F. M. Cimadomo D. Noli L. Khalaf Y. Farcomeni A. (2016). MicroRNAs in spent blastocyst culture medium are derived from trophectoderm cells and can be explored for human embryo reproductive competence assessment. *Fertil. Steril.* 105 225–235.e3. 10.1016/j.fertnstert.2015.09.014 26453979

[B9] Chen H. R. Juan H. C. Wong Y. H. Tsai J. W. Fann M. J. (2017). Cdk12 regulates neurogenesis and late-arising neuronal migration in the developing cerebral cortex. *Cereb. Cortex* 27 2289–2302. 27073218 10.1093/cercor/bhw081

[B10] Chilà R. Guffanti F. Damia G. (2016). Role and therapeutic potential of CDK12 in human cancers. *Cancer Treat. Rev.* 50 83–88. 10.1016/j.ctrv.2016.09.003 27662623

[B11] Dai Q. Lei T. Zhao C. Zhong J. Tang Y. Z. Chen B. (2012). Cyclin K-containing kinase complexes maintain self-renewal in murine embryonic stem cells. *J. Biol. Chem.* 287 25344–25352. 10.1074/jbc.M111.321760 22547058 PMC3408147

[B12] Dinnyes A. (1999) Timing of the first cleavage post-insemination affects cryosurvival of in vitro–produced bovine blastocysts. *Mol. Reprod. Dev.* 53 318–324 10.1002/(SICI)1098-2795(199907)53:3<318::AID-MRD7>3.0.CO;2-O 10369392

[B13] Donker R. B. Mouillet J. F. Chu T. Hubel C. A. Stolz D. B. Morelli A. E. (2012). The expression profile of C19MC microRNAs in primary human trophoblast cells and exosomes. *Mol. Hum. Reprod.* 18 417–424. 10.1093/molehr/gas013 22383544 PMC3389496

[B14] Dweep H. Gretz N. (2015). miRWalk2.0: a comprehensive atlas of microRNA-target interactions. *Nat. Methods* 12:697. 10.1038/nmeth.3485 26226356

[B15] Ekumi K. M. Paculova H. Lenasi T. Pospichalova V. Bösken C. A. Rybarikova J. (2015). Ovarian carcinoma CDK12 mutations misregulate expression of DNA repair genes via deficient formation and function of the Cdk12/CycK complex. *Nucleic Acids Res.* 43 2575–2589. 10.1093/nar/gkv101 25712099 PMC4357706

[B16] Fenwick J. Platteau P. Murdoch A. P. Herbert M. (2002). Time from insemination to first cleavage predicts developmental competence of human preimplantation embryos in vitro. *Hum. Reprod.* 17 407–412. 10.1093/humrep/17.2.407 11821286

[B17] Goossens K. Mestdagh P. Lefever S. Van Poucke M. Van Zeveren A. Van Soom A. (2013). Regulatory microRNA network identification in bovine blastocyst development. *Stem Cells Dev.* 22 1907–1920. 10.1089/scd.2012.0708 23398486 PMC3685315

[B18] Goovaerts I. G. Leroy J. L. Van Soom A. De Clercq J. B. Andries S. Bols P. E. (2009). Effect of cumulus cell coculture and oxygen tension on the in vitro developmental competence of bovine zygotes cultured singly. *Theriogenology* 71 729–738. 10.1016/j.theriogenology.2008.09.038 18962875

[B19] Griffiths-Jones S. Grocock R. J. van Dongen S. Bateman A. Enright A. J. (2006). miRBase: microRNA sequences, targets and gene nomenclature. *Nucleic Acids Res.* 34 D140–D144. 10.1093/nar/gkj112 16381832 PMC1347474

[B20] Gross N. Kropp J. Khatib H. (2017) Sexual dimorphism of miRNAs secreted by bovine in vitro-produced embryos. *Front. Genet.* 8:39. 10.3389/fgene.2017.00039 28421107 PMC5378762

[B21] Gutierrez-Adan A. White C. R. Van Soom A. Mann M. R. (2015). Why we should not select the faster embryo: lessons from mice and cattle. *Reprod. Fertil. Dev.* 27 765–775. 10.1071/RD14216 25209560

[B22] Herrmann D. Dahl J. A. Lucas-Hahn A. Collas P. Niemann H. (2013). Histone modifications and mRNA expression in the inner cell mass and trophectoderm of bovine blastocysts. *Epigenetics* 8 281–289. 10.4161/epi.23899 23406883 PMC3669120

[B23] Hiiragi T. Louvet-Vallée S. Solter D. Maro B. (2006). Does prepatterning occur in the mouse egg?. *Nature* 442:E3. 10.1038/nature04907 16837972

[B24] Huang D. W. Sherman B. T. Lempicki R. A. (2008). Systematic and integrative analysis of large gene lists using DAVID bioinformatics resources. *Nat. Protoc.* 4:44. 10.1038/nprot.2008.211 19131956

[B25] Huang D. W. Sherman B. T. Lempicki R. A. (2009). Bioinformatics enrichment tools: paths toward the comprehensive functional analysis of large gene lists. *Nucleic Acids Res.* 37 1–13. 10.1093/nar/gkn923 19033363 PMC2615629

[B26] Ihaka R. Gentleman R. (1996). R: a language for data analysis and graphics. *J. Comput. Graph. Stat.* 5 299–314.

[B27] Juan H. C. Lin Y. Chen H. R. Fann M. J. (2016). Cdk12 is essential for embryonic development and the maintenance of genomic stability. *Cell Death Differ.* 23 1038–1048. 10.1038/cdd.2015.157 26658019 PMC4987723

[B28] Karbiener M. Neuhold C. Opriessnig P. Prokesch A. Bogner-Strauss J. G. Scheideler M. (2011). MicroRNA-30c promotes human adipocyte differentiation and co-represses PAI-1 and ALK2. *RNA Biol.* 8 850–860. 10.4161/rna.8.5.16153 21878751

[B29] Kropp J. Khatib H. (2015). Characterization of microRNA in bovine in vitro culture media associated with embryo quality and development. *J. Dairy Sci.* 98 6552–6563. 10.3168/jds.2015-9510 26142856

[B30] Li J. Donath S. Li Y. Qin D. Prabhakar B. S. Li P. (2010). miR-30 regulates mitochondrial fission through targeting p53 and the dynamin-related protein-1 pathway. *PLoS Genet.* 6:e1000795. 10.1371/journal.pgen.1000795 20062521 PMC2793031

[B31] Li T. Chen J. X. Fu X. P. Yang S. Zhang Z. Chen Kh H. (2011). microRNA expression profiling of nasopharyngeal carcinoma. *Oncol. Rep.* 25 1353–1363. 21373758 10.3892/or.2011.1204

[B32] Li W. Goossens K. Van Poucke M. Forier K. Braeckmans K. Van Soom A. (2016). High oxygen tension increases global methylation in bovine 4-cell embryos and blastocysts but does not affect general retrotransposon expression. *Reprod. Fertil. Dev.* 28 948–959. 10.1071/RD14133 25515369

[B33] Li X. H. Ha C. T. Fu D. Xiao M. (2012). Micro-RNA30c negatively regulates REDD1 expression in human hematopoietic and osteoblast cells after gamma-irradiation. *PLoS One* 7:e48700. 10.1371/journal.pone.0048700 23144934 PMC3492427

[B34] Liang K. Gao X. Gilmore J. M. Florens L. Washburn M. P. Smith E. (2015). Characterization of human cyclin-dependent kinase 12 (CDK12) and CDK13 complexes in C-terminal domain phosphorylation, gene transcription, and RNA processing. *Mol. Cell. Biol.* 35 928–938. 10.1128/MCB.01426-14 25561469 PMC4333096

[B35] Liu P. (1996). Inactivation of the mouse Brca1 gene leads to failure in the morphogenesis of the egg cylinder in early postimplantation development. *Genes Dev.* 10 1835–1843. 10.1101/gad.10.14.1835 8698242

[B36] Liu X. Li M. Peng Y. Hu X. Xu J. Zhu S. (2016). miR-30c regulates proliferation, apoptosis and differentiation via the Shh signaling pathway in P19 cells. *Exp. Mol. Med.* 48:e248. 10.1038/emm.2016.57 27469029 PMC4973315

[B37] Love M. I. Huber W. Anders S. (2014). Moderated estimation of fold change and dispersion for RNA-seq data with DESeq2. *Genome Biol.* 15:550. 10.1186/s13059-014-0550-8 25516281 PMC4302049

[B38] Lu Z. Zhang C. Han C. An Q. Cheng Y. Chen Y. (2019). Plasticizer bis(2-ethylhexyl) phthalate causes meiosis defects and decreases fertilization ability of mouse oocytes in vivo. *J. Agric. Food Chem.* [Epub ahead of print] 10.1021/acs.jafc.9b00121 30813722

[B39] Luo S. S. Ishibashi O. Ishikawa G. Ishikawa T. Katayama A. Mishima T. (2009). Human villous trophoblasts express and secrete placenta-specific micrornas into maternal circulation via exosomes. *Biol. Reprod.* 81 717–729. 10.1095/biolreprod.108.075481 19494253

[B40] Market Velker B. A. Denomme M. M. Mann M. R. (2012). Loss of genomic imprinting in mouse embryos with fast rates of preimplantation development in culture. *Biol. Reprod.* 86 143 1–16. 22278980 10.1095/biolreprod.111.096602 PMC4480067

[B41] Meirelles F. V. Schwarz K. L. Merighe G. K. F. Carambula S. F. Watanabe Y. F. (2004). 158apoptosis in in vitro produced bovine embryos according to developmental kinetics. *Reprod. Fertil. Dev.* 16 201–201. 10.1071/RDv16n1Ab158

[B42] Milewski R. Ajduk A. (2017). Time-lapse imaging of cleavage divisions in embryo quality assessment. *Reproduction* 154 R37–R53. 10.1530/REP-17-0004 28408705

[B43] Milewski R. Szpila M. Ajduk A. (2018). Dynamics of cytoplasm and cleavage divisions correlates with preimplantation embryo development. *Reproduction* 155 1–14. 10.1530/REP-17-0230 28993454

[B44] Mineno J. Okamoto S. Ando T. Sato M. Chono H. Izu H. (2006). The expression profile of microRNAs in mouse embryos. *Nucleic Acids Res.* 34 1765–1771. 10.1093/nar/gkl096 16582102 PMC1421506

[B45] Mondou E. Dufort I. Gohin M. Fournier E. Sirard M. A. (2012). Analysis of microRNAs and their precursors in bovine early embryonic development. *Mol. Hum. Reprod.* 18 425–434. 10.1093/molehr/gas015 22491901

[B46] Noli L. Capalbo A. Dajani Y. Cimadomo D. Bvumbe J. Rienzi L. (2016). Human embryos created by embryo splitting secrete significantly lower levels of miRNA-30c. *Stem Cells Dev.* 25 1853–1862. 10.1089/scd.2016.0212 27612589 PMC5165679

[B47] Ortiz-Escribano N. Szymańska K. J. Bol M. Vandenberghe L. Decrock E. Van Poucke M. (2017). Blocking connexin channels improves embryo development of vitrified bovine blastocysts^†^. *Biol. Reprod.* 96 288–301. 10.1095/biolreprod.116.144121 28203704

[B48] Ozen M. Creighton C. J. Ozdemir M. Ittmann M. (2007). Widespread deregulation of microRNA expression in human prostate cancer. *Oncogene* 27:1788. 10.1038/sj.onc.1210809 17891175

[B49] Paculová H. Kohoutek J. (2017). The emerging roles of CDK12 in tumorigenesis. *Cell Div.* 12:7. 10.1186/s13008-017-0033-x 29090014 PMC5658942

[B50] Paculová H. Kramara J. Šimečková Š. Fedr R. Souček K. Hylse O. (2017). BRCA1 or CDK12 loss sensitizes cells to CHK1 inhibitors. *Tumor Biol.* 39:1010428317727479. 10.1177/1010428317727479 29025359

[B51] Plusa B. Hadjantonakis A. -K. Gray D. Piotrowska-Nitsche K. Jedrusik A. Papaioannou V. E. (2005). The first cleavage of the mouse zygote predicts the blastocyst axis. *Nature* 434:391. 10.1038/nature03388 15772664

[B52] Poudel S. Song J. Jin E. J. Song K. (2013). Sulfuretin-induced miR-30C selectively downregulates cyclin D1 and D2 and triggers cell death in human cancer cell lines. *Biochem. Biophys. Res. Commun.* 431 572–578. 10.1016/j.bbrc.2013.01.012 23318178

[B53] Quintavalle C. Donnarumma E. Iaboni M. Roscigno G. Garofalo M. Romano G. (2013). Effect of miR-21 and miR-30b/c on TRAIL-induced apoptosis in glioma cells. *Oncogene* 32 4001–4008. 10.1038/onc.2012.410 22964638

[B54] Robinson M. D. McCarthy D. J. Smyth G. K. (2010). edgeR: a Bioconductor package for differential expression analysis of digital gene expression data. *Bioinformatics* 26 139–140. 10.1093/bioinformatics/btp616 19910308 PMC2796818

[B55] Rosenbluth E. M. Shelton D. N. Wells L. M. Sparks A. E. T. Van Voorhis B. J. (2014). Human embryos secrete microRNAs into culture media—a potential biomarker for implantation. *Fertil. Steril.* 101 1493–1500. 10.1016/j.fertnstert.2014.01.058 24786747

[B56] Schwarzenbach H. Nishida N. Calin G. A. Pantel K. (2014). Clinical relevance of circulating cell-free microRNAs in cancer. *Nat. Rev. Clin. Oncol.* 11:145. 10.1038/nrclinonc.2014.5 24492836

[B57] Shi J. Dong M. Li L. Liu L. Luz-Madrigal A. Tsonis P. A. (2015). mirPRo–a novel standalone program for differential expression and variation analysis of miRNAs. *Sci. Rep.* 5:14617. 10.1038/srep14617 26434581 PMC4592965

[B58] Shukla K. Sharma A. K. Ward A. Will R. Hielscher T. Balwierz A. (2015). MicroRNA-30c-2-3p negatively regulates NF-kappaB signaling and cell cycle progression through downregulation of TRADD and CCNE1 in breast cancer. *Mol. Oncol.* 9 1106–1119. 10.1016/j.molonc.2015.01.008 25732226 PMC5528752

[B59] Sohel M. M. Hoelker M. Noferesti S. S. Salilew-Wondim D. Tholen E. Looft C. (2013). Exosomal and non-exosomal transport of extra-cellular microRNAs in follicular fluid: implications for bovine oocyte developmental competence. *PLoS One* 8:e78505. 10.1371/journal.pone.0078505 24223816 PMC3817212

[B60] Sugimura S. Akai T. Imai K. (2017). Selection of viable in vitro-fertilized bovine embryos using time-lapse monitoring in microwell culture dishes. *J. Reprod. Dev.* 63 353–357. 10.1262/jrd.2017-041 28552887 PMC5593086

[B61] Tang F. Kaneda M. O’Carroll D. Hajkova P. Barton S. C. Sun Y. A. (2007). Maternal microRNAs are essential for mouse zygotic development. *Genes Dev.* 21 644–648. 10.1101/gad.418707 17369397 PMC1820938

[B62] Terriou P. Giorgetti C. Hans E. Salzmann J. Charles O. Cignetti L. (2007) Relationship between even early cleavage and day 2 embryo score and assessment of their predictive value for pregnancy. *Reprod. Biomed. Online* 14 294–299. 10.1016/S1472-6483(10)60870-X 17359580

[B63] Tien J. F. Mazloomian A. Cheng S. W. G. Hughes C. S. Chow C. C. T. Canapi L. T. (2017). CDK12 regulates alternative last exon mRNA splicing and promotes breast cancer cell invasion. *Nucleic Acids Res.* 45 6698–6716. 10.1093/nar/gkx187 28334900 PMC5499812

[B64] Valadi H. Ekström K. Bossios A. Sjöstrand M. Lee J. J. Lötvall J. O. (2007). Exosome-mediated transfer of mRNAs and microRNAs is a novel mechanism of genetic exchange between cells. *Nat. Cell Biol.* 9 654–659. 10.1038/ncb1596 17486113

[B65] Van Soom A. (1997) Relationship between timing of development, morula morphology, and cell allocation to innercell mass and trophectoderm *in vitro*-producedbovine embryos. *Mol. Reprod. Dev.* 47 47–56. 10.1002/(SICI)1098-2795(199705)47:1<47::AID-MRD7>3.0.CO;2-Q 9110314

[B66] Vandaele L. Mateusen B. Maes D. de Kruif A. Van Soom A. (2006). Is apoptosis in bovine in vitro produced embryos related to early developmental kinetics and in vivo bull fertility? *Theriogenology* 65 1691–1703. 10.1016/j.theriogenology.2005.09.014 16280159

[B67] Vandaele L. Mateusen B. Maes D. G. de Kruif A. Van Soom A. (2007). Temporal detection of caspase-3 and -7 in bovine in vitro produced embryos of different developmental capacity. *Reproduction* 133 709–718. 10.1530/REP-06-0109 17504915

[B68] Varet H. Brillet-Guéguen L. Coppée J. -Y. Dillies M. -A. (2016). SARTools: a DESeq2- and EdgeR-based R pipeline for comprehensive differential analysis of RNA-seq data. *PLoS One* 11:e0157022. 10.1371/journal.pone.0157022 27280887 PMC4900645

[B69] Vickers K. C. Palmisano B. T. Shoucri B. M. Shamburek R. D. Remaley A. T. (2011). MicroRNAs are transported in plasma and delivered to recipient cells by high-density lipoproteins. *Nat. Cell Biol.* 13 423–433. 10.1038/ncb2210 21423178 PMC3074610

[B70] Vilella F. Moreno-Moya J. M. Balaguer N. Grasso A. Herrero M. Martínez S. (2015). Hsa-miR-30d, secreted by the human endometrium, is taken up by the pre-implantation embryo and might modify its transcriptome. *Development* 142 3210–3221. 10.1242/dev.124289 26395145

[B71] Viswanathan S. R. Mermel C. H. Lu J. Lu C. W. Golub T. R. Daley G. Q. (2009). microRNA expression during trophectoderm specification. *PLoS One* 4:e6143. 10.1371/journal.pone.0006143. 19582159 PMC2702083

[B72] Wong C. C. Loewke K. E. Bossert N. L. Behr B. De Jonge C. J. Baer T. M. (2010). Non-invasive imaging of human embryos before embryonic genome activation predicts development to the blastocyst stage. *Nat. Biotechnol.* 28 1115–1121. 10.1038/nbt.1686 20890283

[B73] Wu H. Zhao J. Fu B. Yin S. Song C. Zhang J. (2017). Retinoic acid-induced upregulation of miR-219 promotes the differentiation of embryonic stem cells into neural cells. *Cell Death Dis.* 8:e2953. 10.1038/cddis.2017.336 28749472 PMC5550877

[B74] Wu T. Zhou H. Hong Y. Li J. Jiang X. Huang H. (2012). miR-30 family members negatively regulate osteoblast differentiation. *J. Biol. Chem.* 287 7503–7511. 10.1074/jbc.M111.292722 22253433 PMC3293535

[B75] Wydooghe E. Heras S. Dewulf J. Piepers S. Van den Abbeel E. De Sutter P. (2014a). Replacing serum in culture medium with albumin and insulin, transferrin and selenium is the key to successful bovine embryo development in individual culture. *Reprod. Fertil. Dev.* 26 717–724. 10.1071/RD13043 23711172

[B76] Wydooghe E. Vandaele L. Piepers S. Dewulf J. Van den Abbeel E. De Sutter P. (2014b). Individual commitment to a group effect: strengths and weaknesses of bovine embryo group culture. *Reproduction* 148 519–529. 10.1530/REP-14-0213 25118302

[B77] Wydooghe E. Vandaele L. Beek J. Favoreel H. Heindryckx B. De Sutter P. (2011). Differential apoptotic staining of mammalian blastocysts based on double immunofluorescent CDX2 and active caspase-3 staining. *Anal. Biochem.* 416 228–230. 10.1016/j.ab.2011.05.033 21684250

[B78] Yang J. Li C. Zhang L. Wang X. (2018). Extracellular vesicles as carriers of non-coding RNAs in liver diseases. *Front. Pharmacol.* 9:415. 10.3389/fphar.2018.00415 29740327 PMC5928552

[B79] Yang Y. Bai W. Zhang L. Yin G. Wang X. Wang J. (2008). Determination of microRNAs in mouse preimplantation embryos by microarray. *Dev. Dyn.* 237 2315–2327. 10.1002/dvdy.21666 18729214

[B80] Zernicka-Goetz M. (2002). Patterning of the embryo: the first spatial decisions in the life of a mouse. *Development* 129:815. 11861466 10.1242/dev.129.4.815

[B81] Zernicka-Goetz M. (2006). The first cell-fate decisions in the mouse embryo: destiny is a matter of both chance and choice. *Curr. Opin. Genet. Dev.* 16 406–412. 10.1016/j.gde.2006.06.011 16806896

