# Supplementary material for: Bovine Embryo-Secreted microRNA-30c Is a Potential Non-invasive Biomarker for Hampered Preimplantation Developmental Competence
Source: Front Genet. 2019 Apr 5;10:315. doi: 10.3389/fgene.2019.00315 (PMC6459987; doi:10.3389/fgene.2019.00315)
Supplement: Supplementary file 1 [file Table_1.docx]

Supplemental_Table_S1 **Sequences of primers used in this study**

| Name | Sequence (5’- 3’) |
| --- | --- |
| psiCHECK2-CDK12 3'UTR vector construction |  |
| BtauCDK12 3'utr-F | tttatttatttCTCGAGGGACATTGCTGCTGAGGTAGGG |
| BtauCDK12 3'utr-R | tttatttatttGCGGCCGCTGTCAATACTGTTCTTTCTTATGGGATACTCT |
| BtauCDK12 3'utr mut-R1 (mutations underlined) | TATGTGTAAACAGTATGCTAACTGATGTTA |
| BtauCDK12 3'utr mut-F1(mutations underlined) | TGGAGGGTTAGTAACATCAGTTAGCATACTGTTTAC |
| pEGFP-N1-CDK12 vector construction (CDK12 CDS) |  |
| Primer 1 (product length 2469 ) |  |
| F1-F | GAGTGCTGGGGAACTTTT |
| F1-R | GGCTCCTTTGTCCTTCTTG |
| Primer 2 (product length 2292) |  |
| F2-F | TGTCACAGATAAACAGGATG |
| F2-R | GAGGAACTGGTGCCAATAT |
| Primer with restriction site (product length 4473) |  |
| BtauCDK12-Nhe1-F | AATCGCTAGCAATGCCCAATCCAGAG |
| BtauCDK12-Xho1-R | CTAACTCGAGTCTAGGGTAAGGAACTCCTC |
| CDK12 mRNA level |  |
| BtauCDK12-F | CAGGGAAAGTGAAGTTGG |
| BtauCDK12-R | GGGGTAGTAGTTGGTAAAGG |
| BtauGAPDH-F | TTCAACGGCACAGTCAAGG |
| BtauGAPDH-R | ACATACTCAGCACCAGCATCAC |
| DDR genes |  |
| BtauBRCA1-F | GAGTTTGTGTGTGAACGGACACTGA |
| BtauBRCA1-R | CCACTCTAGTTGATCTGTGGGCATATTG |
| BtauFANCD2-F | AAACCAGCACGGGCACATCTT |
| BtauFANCD2-R | TATCCACAACTCGGTCCAGCCA |
| BtauFANCI-F | CCCATCATTCTCACTGCCCTTGGT |
| BtauFANCI-R | TTCCGGCTTCCCTTTGAGGAGAGA |
| BtauATR-F | ACAGGAAGACCTCAGCAGTAATAGCA |
| BtauATR-R | ACAGTCCTTGAAATTATAGCAGTCCATGTT |
